# Effects of alpha-linolenic acid *vs*. docosahexaenoic acid supply on the distribution of fatty acids among the rat cardiac subcellular membranes after a short- or long-term dietary exposure

**DOI:** 10.1186/1743-7075-6-14

**Published:** 2009-03-25

**Authors:** Amandine Brochot, Marine Guinot, Daniel Auchere, Jean-Paul Macaire, Pierre Weill, Alain Grynberg, Delphine Rousseau-Ralliard

**Affiliations:** 1 Institut National de la Recherche Agronomique (INRA)-Université Paris-Sud 11, Unité Mixte de Recherche 1154, Lipides Membranaires et Régulation Fonctionnelle du Coeur et des Vaisseaux, Institut Fédératif de Recherche 141, Faculté de Pharmacie, Châtenay-Malabry, F-92296, France; 2 Société Valorex, Combourtillé, France

## Abstract

**Background:**

Previous work showed that the functional cardiac effect of dietary alpha-linolenic acid (ALA) in rats requires a long feeding period (6 months), although a docosahexaenoic (DHA) acid-supply affects cardiac adrenergic response after 2 months. However, the total cardiac membrane n-3 polyunsaturated fatty acid (PUFA) composition remained unchanged after 2 months. This delay could be due to a specific reorganization of the different subcellular membrane PUFA profiles. This study was designed to investigate the evolution between 2 and 6 months of diet duration of the fatty acid profile in sarcolemmal (SL), mitochondrial (MI), nuclear (NU) and sarcoplasmic reticulum (SR) membrane fractions.

**Methods:**

Male Wistar rats were randomly assigned to 3 dietary groups (n = 10/diet/period), either n-3 PUFA-free diet (CTL), or ALA or DHA-rich diets. After 2 or 6 months, the subcellular cardiac membrane fractions were separated by differential centrifugations and sucrose gradients. Each membrane profile was analysed by gas chromatography (GC) after lipid extraction.

**Results:**

As expected the n-3 PUFA-rich diets incorporated n-3 PUFA instead of n-6 PUFA in all the subcellular fractions, which also exhibited individual specificities. The diet duration increased SFA and decreased PUFA in SL, whereas NU remained constant. The SR and MI enriched in n-3 PUFA exhibited a decreased DHA level with ageing in the DHA and CTL groups. Conversely, the n-3 PUFA level remained unchanged in the ALA group, due to a significant increase in docosapentaenoic acid (DPA). N-3 PUFA rich diets lead to a better PUFA profile in all the fractions and significantly prevent the profile modifications induced by ageing.

**Conclusion:**

With the ALA diet the n-3 PUFA content, particularly in SR and SL kept increasing between 2 and 6 months, which may partly account for the delay to achieve the modification of adrenergic response.

## Background

Cardiac phospholipids are known to be organized into functionally differentiated domains providing both structural integrity and a suitable microenvironment for optimization of membrane protein function [1]. Furthermore, both n-6 and n-3 polyunsaturated fatty acids (PUFA) are important structural and functional components of cell membrane phospholipids. An important source of dietary n-3 PUFA is α-linolenic acid (ALA, 18:3 n-3), supplied by vegetable sources (linseed, rapeseed, soybean, nuts), whereas the longer chain n-3 PUFA (n-3 LCPUFA), mainly eicosapentaenoic acid (EPA, 20:5 n-3) and docosahexaenoic acid (DHA, 22:6 n-3), are supplied by marine products [2]. As reported in the literature n-3 PUFA are known to display pleiotropic effects, and more particularly in the prevention of cardiovascular disease (see for review [3-5]). The beneficial cardiovascular effects of dietary n-3 PUFA have been largely attributed to the long chain components (n-3 LCPUFA) and this addresses the question of the rapid decrease in the marine sources [2]. Feeding humans or animals with vegetable sources of n-3 LCPUFA precursor such as ALA-rich flaxseed flour could be an alternative, but the role of ALA on cardiovascular function and cardio-metabolic risk remains a matter of debate. This controversy is mainly based on the fact that an increased consumption of ALA results in a slight increase in EPA concentration in plasma and blood cells, but a dramatically low conversion to DHA in humans [6,7]. The production of long chains from ALA appears to be somewhat better in rats [8,9], but depends largely on the tissue considered [8,10]. We have shown recently that if ALA can decrease triglyceridemia, only n-3 LCPUFA prevent significantly the onset of insulin resistance in fructose-fed rats [11]. The incorporation of n-3 PUFA was reported to influence cardiac β-adrenergic response in cardiac cells [12] and heart rate *in vivo *as reported in rat [13] and man [14]. In a previous study, we reported a decrease in heart rate and an increased responsiveness to β-adrenergic stimulation in DHA-fed rats after 2 months [8]. Interestingly, a similar effect was observed with an ALA-rich diet but only after a 6-month feeding period [8]. This delay in the onset of functional effect cannot be attributed only to the maximal incorporation of DHA in whole heart membranes that was achieved at most in 2 months with either the DHA- or the ALA-rich diets. The present study was designed to evaluate the hypothesis of a particular rearrangement of the distribution of n-3 PUFA among the different subcellular membranes, depending on the quality of the dietary n-3 PUFA supplied and the duration of food supply. Thus nuclear (NU), sarcolemmal (SL), mitochondrial (MI) and sarcoplasmic reticulum (SR) membranes were separated in the myocardium of rats fed for 2 or 6 months a diet containing either DHA (from fish oil) or ALA (from linseed flour) to analyse their fatty acid profiles in order to explain the time-related functional effect observed with dietary DHA and ALA.

## Materials and methods

### Animals and diets

The protocol complies with the institutional guidelines for animal research (NIH Pub. No. 85-23, National Research Council, Revised 1996) and was approved by the Animal Care and Use Committee of the Faculty of Pharmacy, University of Paris-Sud 11. The animal facilities are registered under agreement number n°A 92-019-01 and the main authors are fully authorized to manage experiments on animal (agreement level I, ref. 92–261, 2006–09-12). Five week old male Wistar-Han rats (175 ± 10 g, Charles River, L'Abresle, France) were housed grouped by 3 or 4 and maintained at 23 ± 1°C, with a 12-h light/dark cycle. After a 6-day acclimation period on a standard diet (Safe A04, Villemoisson-sur-Orge, France), the rats were randomly assigned to 3 dietary experimental groups for either a 2- or a 6-month feeding period (n = 10/diet/period). These experimental diets were designed as an n-3 PUFA-free diet (CTL), a DHA-rich diet (DHA), or an ALA-rich diet (ALA). These diets were manufactured by UPAE-INRA (INRA, Jouy-en-Josas, France) (Table 1). All diets were designed to bring 80 g.kg^-1 ^of lipids and similar amount of non-lipid components. The ALA diet was based on extruded linseed flour (Valomega 160™, Valorex, Combourtillé, France) partly at the expense of the regular ingredients used in the other diets (Table 1). In the ALA diet, a part of the lipids was thus not brought as oil. However, it was shown that ingestion of both flax oil and milled flaxseed deliver significant and similar levels of ALA to the plasma, and that milled flaxseed did not induce the adverse gastrointestinal effects observed with oil preparations [15]. The fatty acid profile of each experimental diet is presented in Table 1.

**Table 1:** Formulation and fatty acid composition of the experimental diets.

	**CTL diet**	**DHA diet**	**ALA diet**	**Extruded linseed flour^5^**
	g/kg of diet	g/kg of diet	g/kg of diet	g/kg
**Basal mix^1^**				
Protein				200
Soy protein isolate^2^	170	170	147	
Glucides				110
Sucrose	220	220	216	35
Cornstarch	440	440	402	
Fibers (mucilages, ...)				171
Cellulose	20	20		80
**Minerals and other components**				44
L-Cystine	5	5	5	
Choline chloride	5	5	5	
Mineral mixture^3^	50	50	48	
Vitamin mixture^3^	10	10	10	
**Extruded linseed flour^4^**			**122**	
**Lipids**				280
hydrogenated coconut oil^5^	15.2	15	11.3	
Cocoa butter^6^	14.4	18	25.7	
Sunflower seed oil^7^	48	17	8.9	
Rapeseed oil^8^	2.4	10		
n-3 LCPUFA-rich oil^9^		20		
Humidity				80

**Fatty acid composition^10^**	% of total FA	% of total FA	% of total FA	% of total FA
14:0	4.7	4.6	3.5	-
16:0	11.2	13.2	10.2	5.9
18:0	8.5	11.4	8.4	2.9
18:1 n-9	21.7	17.5	17.0	17.3
18:2 n-6	35.5	16.9	18.2	17.7
18:3 n-3	0.6	23.3	1.4	55.1
20:5 n-3	-	-	2.5	-
22:2 n-6	0.3	0.5	0.5	-
22:5 n-3	-	-	16.8	-
22:6 n-3	40.6	40.7	39.8	-
Total SFA	40.6	40.7	39.8	9.1
Total MUFA	22.7	18.4	18.0	18.1
Total PUFA	36.8	40.8	42.2	72.8
Total n-6 PUFA	36.0	17.5	20.5	17.7
Total n-3 PUFA	0.7	23.4	21.7	55.1
n-6/n-3 ratio	50.6	0.7	0.9	0.3
PUFA/SFA ratio	0.9	1.0	1.1	8.0

SFA. saturated fatty acids; MUFA. monounsaturated fatty acids. PUFA. polyunsaturated fatty acids; LCPUFA. long chain PUFA.

^1^. The basal mix components in g/kg of diet were home-made as pellets by the UPAE (INRA). based on a formulation adapted from the AIN-93 (56).

^2^. MP Biomedicals 905456.

^3^. Mineral AIN-93M and Vitamin AIN-93VX mixtures were prepared according to AIN-93 (56). MP Biomedicals.

^4^. Valomega 160™. Valorex. Combourtillé. France

^5^. Vegetaline. SCO. France.

^6^. Barry Callebaut. Meulan. France

^7^. Fruidor. Lesieur. Antony. France

^8^. Rustica. France

^9^. Long chain n-3 PUFA-enriched oil. V0137. Laboratoire Pierre Fabre. France

^10^. As determined by the analysis of the manufactured experimental diets and of the experimental extruded linseed flour.

### Subcellular membrane preparation

At the end of the 2 or 6 month feeding periods, the rats were anesthetised by intraperitoneal pentobarbital injection (40 mg/kg) and killed by heart removal. Quickly dissected, the hearts were washed in ice-cold HSEP buffer (50 mM Hepes, 0.30 M sucrose, 1 mM EDTA and 0.1 mM PMSF) and immediately frozen in liquid nitrogen and stored at -80°C. For biochemical investigation, the hearts were thawed, rinsed with HSEP buffer, cut in small pieces in 10 volumes of HSEP buffer and homogenised using a polytron (three 10-s bursts separated by 30-s at setting 9). The homogenate was fractionated according to Paris *et al*. [16] with slight modifications. Briefly, the homogenate was filtrated through cheesecloth and centrifuged (7 min, 2000 g). The pellet (P1) was re-suspended with 10 mL of HSEP with a Stirrer Type BS Potter-Elvehjem and the suspension (SN1) was centrifuged again (5 min, 2000 g). The pellet (P2) was the nuclear membrane enriched fraction (NU). Both supernatants were pooled (SN2) and centrifuged (11000 g). The pellet (P3) was re-suspended in 10 mL HSEP and centrifuged again in the same conditions. The residual pellet (P4) was the mitochondria enriched fraction and the supernatants, distributed in several tubes, were centrifuged (1 h, 70 000 g) to collect the microsomal enriched fraction. According to Lombet *et al*. [17], the microsomal fraction was distributed in 3 parts charged on the top of 3 discontinuous sucrose density gradients (8 mL 35% sucrose, 14 mL 27% sucrose and 10 mL 15% sucrose) and centrifuged (2 h, 23000 g). The sarcolemma enriched fraction (SL) was collected at the 15%/27% interface and the sarcoplasmic reticulum enriched fraction (SR) at the 27%/35% interface. All the fractions were collected in 10 mL of HEP buffer (50 mM Hepes, 1 mM EDTA and 0.1 mM PMSF) by centrifugation (1 h, 34000 g) and kept at -80°C in 0.5 mL of HEP buffer.

### Western Blot

The membrane fractions were homogenized in lysis buffer (150 mM NaCl, 50 mM Tris, 1 mM EDTA, 1 mM PMSF, 1% Sigma inhibitor cocktail), and the protein content was determined using the Sigma Bicinchoninic Acid Kit for protein determination. Fraction proteins of lysates (20 μg) were separated on NuPAGE 4–12% Bis-Tris gels (Invitrogen) and transferred onto a nitrocellulose membrane. The membranes were blocked with 5% non-fat dry milk and incubated overnight at 4°C with primary antibodies against monoclonal Na/K ATPase (diluted 1/5000, ab7671, Abcam, Cambridge, England), rabbit polyclonal Cox-2 (M-19), goat polyclonal Serca2 (C-20) or rabbit polyclonal Lamin A protein (H-102) (diluted 1/500, references sc-1747, sc-8094 and sc-20680, respectively, Santa-Cruz, Heidelberg, Germany) as sarcolemmal, mitochondrial, sarcoplasmic reticulum and nuclear markers, respectively. Secondary specific horseradish-peroxidase linked antibodies (diluted 1/5000, 1/10000 and 1/2500 for the rabbit, goat and mouse secondary antibodies respectively) were added for 2 h at room temperature. Bound antibodies were detected using the enhanced chemiluminescence system (ECL, Amersham Bioscience) with Kodak Biomax light films. The bands of immunoblots were scanned and quantified by densitometric analysis using Scion Image software (Scion Corp., Frederick, MD).

### Lipid analysis

The lipids were extracted with chloroform/methanol (2/1) according to an adaptation of the method of Folch and Lees [18] as previously described [8]. After extraction, the fatty acids, mostly contained in membrane phospholipids, were transmethylated with Boron trifluoride methanol 7% (Sigma-aldrich, Saint Quentin Fallavier, France) according to Morrisson *et al*. [19]. As previously described [13], the methyl esters of phospholipid fatty acids were analyzed by gas chromatography coupled to FID (Auto Sampling 8410 Gas Chromatograph 3900; Varian, Les Ulis, France) on an Econo-Cap EC-WAX capillary column (30-m, 0.32-mm internal diameter, 0.25-μm Film, ref 19654, ALLTECH Associates Inc, Templemars, France), using heptadecanoic acid (margaric acid, C17:0) as internal standard. Hydrogen was used as the carrier gas. The Unsaturation Index (UI) was calculated as UI = sum (% FA × number of double bound).

### Glycaemia, triglycerides and cholesterol assays

EDTA-plasma total cholesterol, triglyceride and glucose levels were determined enzymatically on Modular-Roche automate (Roche, Meylan, France).

### Statistical analysis

The data were expressed as means ± SEM, n = 10. The data obtained from each group after a 2- or 6-month period of dietary treatment were evaluated by a 2-way ANOVA with dietary groups and duration of dietary treatment as fixed factors. When significantly different (P < 0.05), the means were compared with the Newman-Keuls test. All statistical analyses were conducted using NCSS 2000 software.

## Results

### Effect of dietary manipulations on plasma fatty acid profile and lipids

The weight gain with time was similar in the 3 dietary groups (data not shown). Glycaemia was similar whatever the diet. (11.4–12.5 g/L).

#### Plasma lipids

Plasma total cholesterol was decreased in the DHA group (1.2 ± 0.03 g/L, p < 0.01) but remained unaffected in the 2 other groups (1.57 ± 0.08 and 1.58 ± 0.08 g/L in CTL and ALA groups, respectively). Plasma triglyceride level significantly decreased after 1 month in both DHA and ALA groups and remained lower thereafter. This decrease was significantly more pronounced in the DHA group than in the ALA group (1.39 ± 0.11 and 1.83 ± 0.12 g/L, respectively, p < 0.01) as compared to CTL group (2.25 ± 0.2504 g/L).

#### Plasma fatty acids

The quality of the dietary fatty acids significantly affected the plasma PUFA. In the DHA-fed rats, DHA increased largely and eicosapentaenoic acid (EPA, 20:5 n-3) and docosapentaenoic acid (DPA, 22:5 n-3) increased in a lower extent. In the ALA-fed rats, the main increase concerned ALA, and EPA and DPA increased in a lower extent. These n-3 PUFA increases occurred mostly at the expense of arachidonic acid (AA, 20:4n-6) (Table 2). With time, whatever the diet (CTL, ALA or DHA), both n-3 and n-6 PUFA content decreased in plasma fatty acids, affecting each individual PUFA and proportionally more the n-3 PUFA, leading to an increased n-6/n-3 ratio. This decrease with time of PUFA was parallel associated with a SFA increase (mainly palmitic acid, 16:0), leading to a decreased PUFA/Saturated fatty acid (PUFA/SFA, P/S) ratio. Moreover in the two n-3 PUFA-fed groups, the plasma fatty acid concentration was lower as compared to the CTL-fed rats in agreement with the plasma triglyceride content as shown above. With time, the plasma fatty acid concentration increased significantly in CTL-fed rats, but remained low in the two n-3 PUFA-fed groups.

**Table 2:** Plasma fatty acid profiles (% of total fatty acids) from rats fed experimental diets for 2 or 6 months.

Duration (months)	2	2	2	6	6	6	
Dietary groups	CTL	DHA	ALA	CTL	DHA	ALA	

Fatty acids:							CI
14:0	0.6 ± 0.6	0.2 ± 0.1	0.2 ± 0.0	1.0 ± 0.2	1.0 ± 0.2***	0.8 ± 0.1	-
16:0	14.6 ± 1.3^b^	17.3 ± 1.4^a^	17.6 ± 0.8^a^	20.0 ± 0.8^b ^***	24.2 ± 0.8^a ^***	22.3 ± 0.8^a ^***	-
16:1 n-9	0.2 ± 0.1	0.2 ± 0.0	0.2 ± 0.0	0.3 ± 0.0	0.3 ± 0.1***	0.4 ± 0.0	-
16:1 n-7	1.7 ± 0.1	2.0 ± 0.6	2.2 ± 0.4	2.4 ± 0.8	2.7 ± 0.7	2.4 ± 0.8	-
18:0	12.1 ± 1.0^b^	12.2 ± 0.5^b^	13.7 ± 1.0^a^	12.3 ± 0.9^b^	13.2 ± 1.0^b^	14.5 ± 0.8^a^	-
18:1 n-9	15.4 ± 0.9	16.8 ± 0.9	15.5 ± 1.0	15.4 ± 1.2*	14.9 ± 2.1*	13.9 ± 0.5*	-
18:1 n-7	2.1 ± 0.2	1.8 ± 0.1	1.9 ± 0.2	2.2 ± 0.4^a^	1.7 ± 0.2^b^	1.6 ± 0.1^b^	-
18:2 n-6	27.3 ± 1.4^a^	19.3 ± 1.4^b^	20.3 ± 1.0^b^	25.5 ± 1.3^a ^*	19.0 ± 0.9^b^	18.7 ± 0.9^b^	-
18:3 n-6	0.4 ± 0.1^a^	0.3 ± 0.1^a^	<0.1^b^	0.5 ± 0.1^a^	<0.1^c^	0.2 ± 0.1^b^	-
18:3 n-3	0.3 ± 0.0^b^	0.7 ± 0.1^b^	8.4 ± 0.9^a^	0.3 ± 0.0^b^	0.7 ± 0.1^b^	8.1 ± 0.4^a^	-
20:3 n-6	0.5 ± 0.1^b^	0.4 ± 0.1^b^	0.7 ± 0.2^a^	0.5 ± 0.1^a^	0.4 ± 0.1^b^	0.6 ± 0.1^a^	-
20:4 n-6	21.3 ± 0.6^a^	7.4 ± 0.9^b^	7.5 ± 0.6^b^	16.7 ± 1.7^a ^***	6.7 ± 1.0^b^	6.7 ± 0.5^b^	-
20:5 n-3	<0.1^b^	5.7 ± 0.8^a^	5.6 ± 0.8^a^	<0.1^c^	3.4 ± 0.4^b ^***	4.8 ± 0.3^a ^***	$
22:4 n-6	0.5 ± 0.1^a^	<0.1^b^	<0.1^b^	0.4 ± 0.1^a^	<0.1^b^	<0.1^b^	-
22:5 n-6	0.5 ± 0.1^a^	0.5 ± 0.0^a^	<0.1^b^	0.4 ± 0.1^a^	0.1 ± 0.01^a^	<0.1^b^	-
22:5 n-3	0.1 ± 0.0^c^	1.0 ± 0.2^b^	1.8 ± 0.2^a^	<0.1^c^	0.6 ± 0.1^b ^***	1.4 ± 0.2^a ^***	$
22:6 n-3	0.8 ± 0.1^c^	12.6 ± 0.7^a^	2.8 ± 0.4^b^	0.6 ± 0.1^c^	9.3 ± 0.4^a ^***	2.2 ± 0.3^b^	$

SFA	27.8 ± 1.7^b^	30.3 ± 1.4^a^	32.0 ± 1.0^a^	33.8 ± 0.5^b ^***	38.9 ± 1.4^a ^***	38.1 ± 0.6^a ^***	-
MUFA	19.9 ± 1.1	21.2 ± 1.6	20.2 ± 1.5	20.7 ± 2.1	19.9 ± 2.2	18.6 ± 0.8	-
PUFA	52.2 ± 0.7^a^	48.5 ± 2.6^b^	47.8 ± 1.1^b^	45.5 ± 2.1^a ^***	41.2 ± 1.8^b ^***	43.3 ± 0.6^b ^***	-
n-6 PUFA	50.8 ± 0.8^a^	28.4 ± 2.1^b^	28.8 ± 1.6^b^	44.3 ± 2.0^a ^***	27.2 ± 1.6^b^	26.4 ± 0.9^b^	$
n-3 PUFA	1.3 ± 0.1^b^	20.1 ± 1.5^a^	18.9 ± 1.1^a^	1.1 ± 0.1^b^	14.0 ± 0.6^a ^***	16.8 ± 0.7^a ^***	$
n-6/n-3 ratio	37.9 ± 2.8^a^	1.4 ± 0.2^b^	1.5 ± 0.2^b^	40.1 ± 1.2^a ^*	1.9 ± 0.1^b ^*	1.6 ± 0.1^b ^*	-
P/S ratio	1.9 ± 0.1^a^	1.6 ± 0.2^b^	1.5 ± 0.1^b^	1.3 ± 0.1^a ^***	1.1 ± 0.1^b ^***	1.1 ± 0.0^b ^***	-

[plasma FA] (μg/mL)	803 ± 6.8^a^	427 ± 49.7^b^	590 ± 35.2^b^	858 ± 14.4^a ^*	441 ± 67.2^b^	519 ± 32.4^b^	$

SFA. MUFA. and PUFA. saturated. monounsaturated. and polyunsaturated FA; CTL. n-3 PUFA free diet; DHA. docosahexaenoic acid-rich diet; ALA. alpha linolenic acid-rich diet.

Data are means ± SEM. n = 4. Dietary effect (CTL versus DHA versus ALA) was compared in rats from the same dietary treatment duration and values with different letters are statistically different. a>b>c. p < 0.01. Time effect (2 months versus 6 months) was compared between rats from the same dietary group. with * = p < 0.05; ** = p < 0.01. *** = p < 0.001). Symbol $ denoted significant cross-interaction. p < 0.05.

### Membrane fractionation

**Figure 1:**
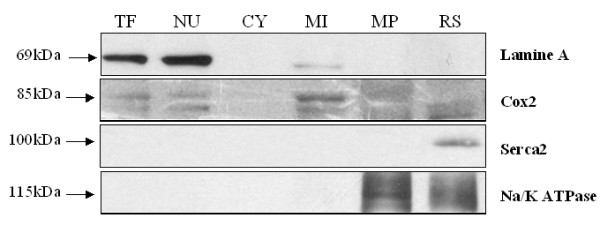
**Specific protein markers of the different enriched fraction by Western-blotting**. Membrane fraction were lysed in a RIPA buffer (see material and method for the composition) and 20 μg of the lysate was used for electrophoresis. Blots from Serca2 and Na/KATPase were made on the same membrane after 30 min of deshybridation at 50°C with a stripping buffer (100 mM mercapto-ethanol, 2% SDS and 62.5 mL Tris-HCl, pH 6.7). Cox-2 and Lamin A were made also on a same membrane. Western blot analysis shows single protein bands of 69 kDa, 85 kDa 115 kDa and 100 kDa for Lamine A, COX-2, Na/K ATPase and Serca2 respectively in rat heart fractions. Each specific protein band was observed in the specific fraction with a slight contamination of the NU in the MI fraction and a slight presence of the MI fraction in the NU fraction. TF, total fraction; NU, nucleus membrane fraction, CY, cytosolic fraction; MI, mitochondrial membrane fraction; SL, sarcolemmal membrane fraction; SR, sarcoplasmic reticulum fraction.

Western-Blotting analysis showed that the different fraction analysed were well-separated. No contamination from membrane fractions was observed in the cytosolic fraction (Figure 1). We observed a very slight contamination of the NU and MI fractions by the MI and the NU fractions, respectively. The SL, NU, MI and SR fractions were enriched by 90%, 35%, 50% and 90% respectively as compared to the total fraction as evaluated by densitometry analyses.

### Effect of dietary manipulations on cardiac subcellular membrane fatty acid profile

#### Whole heart

There was no significant difference in total fatty acid content of each membrane fraction, whatever the dietary treatment. So, presentations of fatty acid profiles were limited to the expression of each fatty acid as percentage of total fatty acids. In the whole heart phospholipids (Table 3), the dietary-induced fatty acid profile changes were similar to those reported in our previous study [8]. The proportions of SFA, monounsaturated fatty acids (MUFA) and PUFA were weakly influenced by diet or duration. The n-6 PUFA and n-3 PUFA contents were significantly affected by the diet but not significantly affected by the time, without any significant cross-interaction between these 2 factors. The n-6 PUFA content in the total cardiac phospholipids was 52 ± 1.0% in the CTL group and decreased significantly (p < 0.01) with n-3 PUFA intake after 2 months (39 ± 1.3% and 31 ± 1.3% in the ALA and DHA groups, respectively), and remained unchanged thereafter. Conversely, the n-3 PUFA content in cardiac phospholipids was 5.4 ± 0.5% in the CTL group and increased significantly (p < 0.01) with n-3 PUFA intake after 2 months of diet (17.5 ± 1.5% and 26.0 ± 0.7% in the ALA and DHA groups, respectively) and did not change significantly thereafter. This increase in n-3 PUFA concerned mainly the DHA in the DHA group (23–24% of the 26% of total n-3). Conversely, in the ALA group, DHA represented only 10% of the 17% of total n-3) with DPA accounting for 4–5%. EPA slightly increased in the cardiac phospholipids of the rats fed either DHA or ALA, but never exceeded 1% or 1.5%, respectively. Cardiac ALA increased only in the ALA group, but never exceeded 1%. These increases in n-3 PUFA occurred mainly at the expense of the arachidonic acid (20:4 n-6, AA). Moreover, we did not observe significant changes in whole heart membrane profile between 2 and 6 months of diet, except the increase in AA in the CTL group and an increase of linoleic acid (18:2 n-6, LA) in the ALA group. Thus, the most significant shift between groups was the proportion of n-6 PUFA and n-3 PUFA (p < 0.001) induced by n-3 PUFA dietary intake, as compared to the CTL group, evidenced by a decrease in the n-6/n-3 ratio more pronounced in the DHA group than in the ALA group.

**Table 3:** Cardiac phospholipids fatty acid profiles (% of total fatty acids) from rats fed experimental diets for 2 or 6 months.

Duration (months)	2	2	2	6	6'	6	
Dietary groups	CTL	DHA	ALA	CTL	DHA	ALA	

Fatty Acids							CI
16:0	9.8 ± 0.56	11.5 ± 0.52	11.2 ± 0.78	10.0 ± 0.76	12.3 ± 0.42	11.4 ± 0.49	-
18:0	24.9 ± 0.38	24.7 ± 0.32	25.5 ± 0.43	25.5 ± 0.48	24.7 ± 0.47	25.0 ± 0.36	-
18:1 n-9	3.5 ± 0.27	3.2 ± 0.15	3.6 ± 0.30	3.5 ± 0.14	3.2 ± 0.30	3.8 ± 0.09	-
18:1 n-7	2.9 ± 0.24^a^	2.2 ± 0.06^b^	2.6 ± 0.13^ab^	2.7 ± 0.11	1.9 ± 0.26	2.7 ± 0.07	-
18:2 n-6	20.5 ± 2.32	17.2 ± 1.39	20.9 ± 1.18	19.7 ± 1.63^ab^	17.8 ± 0.65^b^	22.7 ± 0.40^a^	-
18:3 n-3	<0.1^b^	<0.1^b^	0.8 ± 0.05^a^	<0.1^b^	<0.1^b^	0.9 ± 0.04^a^	-
20:4 n-6	24.6 ± 0.66^a^	12.3 ± 0.32^c^	16.2 ± 0.46^b^	26.6 ± 0.73^a ^*	12.2 ± 0.26^c^	15.4 ± 0.32^b^	$
20:5 n-3	<0.1^c^	0.9 ± 0.05^b^	1.4 ± 0.06^a^	<0.1^c^	0.9 ± 0.05^b^	1.5 ± 0.06^a^	-
22:4 n-6	1.7 ± 0.23^a^	<0.1^b^	<0.1^b^	1.5 ± 0.04^a^	<0.1^b^	<0.1^b^	-
22:5 n-6	3.9 ± 0.61^a^	0.8 ± 0.03^b^	<0.1^c^	3.6 ± 0.07^a^	0.7 ± 0.01^b^	<0.1^c^	
22:5 n-3	0.7 ± 0.05^b^	0.8 ± 0.04^b^	4.5 ± 0.46^a^	0.8 ± 0.06^b^	0.7 ± 0.03^b^	4.3 ± 0.16^a^	-
22:6 n-3	4.6 ± 0.45^c^	24.2 ± 0.64^a^	10.3 ± 1.06^b^	4.0 ± 0.32^c^	23.4 ± 0.80^a^	9.8 ± 0.83^b^	-

SFA	35.6 ± 0.72	36.9 ± 0.70	37.3 ± 1.20	36.3 ± 1.26	37.8 ± 0.84	37.2 ± 0.83	-
MUFA	7.0 ± 0.49	6.1 ± 0.16	7.0 ± 0.48	6.9 ± 0.20^ab^	5.8 ± 0.27^b^	7.4 ± 0.21^a^	-
PUFA	57.5 ± 0.81	57.0 ± 0.61	55.7 ± 1.62	56.8 ± 1.17	56.4 ± 0.89	55.4 ± 1.01	-
n-6 PUFA	52.1 ± 0.91^a^	31.0 ± 1.25^c^	38.5 ± 1.28^b^	51.9 ± 0.89^a^	31.2 ± 0.64^c^	38.8 ± 0.24^b^	-
n-3 PUFA	5.4 ± 0.49^c^	26.0 ± 0.69^a^	17.1 ± 1.47^b^	4.8 ± 0.37^c^	25.2 ± 0.77^a^	16.5 ± 0.87^b^	-
LA/AA ratio	0.8 ± 0.11^b^	1.4 ± 0.14^a^	1.3 ± 0.11^a^	0.7 ± 0.08^b^	1.5 ± 0.06^a^	1.5 ± 0.06^a^	-
AA/DHA ratio	5.5 ± 0.48^a^	0.5 ± 0.02^c^	1.6 ± 0.17^b^	6.8 ± 0.73^a ^*	0.5 ± 0.02^c^	1.6 ± 0.15^b^	-
n-6/n-3 ratio	10.0 ± 1.08^a^	1.2 ± 0.08^c^	2.3 ± 0.21^b^	10.8 ± 0.73^a^	1.2 ± 0.05^c^	2.4 ± 0.12^b^	-
P/S ratio	0.6 ± 0.02	0.6 ± 0.02	0.7 ± 0.04	0.6 ± 0.04	0.7 ± 0.03	0.7 ± 0.03	-

SFA. MUFA. and PUFA. saturated. monounsaturated. and polyunsaturated FA; CTL. n-3 PUFA free diet; DHA. docosahexaenoic acid-rich diet; ALA. alpha linolenic acid-rich diet.

Data are means ± SEM. n = 6. Dietary effect (CTL versus DHA versus ALA) was compared in rats from the same dietary treatment duration and values with different letters are statistically different. a>b>c. p < 0.01. Time effect (2 months versus 6 months) was compared between rats from the same dietary group. with * = p < 0.05; ** = p < 0.01. *** = p < 0.001). Symbol $ denoted significant cross-interaction. p < 0.05.

#### SL fraction

After 2 months of experimental diet, SL exhibited a highly unsaturated fatty acid profile with a total PUFA content of 52–58%, SFA and MUFA representing approximately 32–39% and 8–10%, respectively (Table 4). The SL fatty acid profile changed with dietary n-3 PUFA intake that induced a significant increase in DHA mostly in the DHA group whereas EPA, DPA and DHA (in a lower extent) increased in the ALA group. These changes mostly occurred at the expense of AA and longer chain end-products of the n-6 PUFA series (22:4 n-6 and 22:5 n-6). DHA content was increased 5 fold and 2 fold in the DHA and ALA groups, respectively, as compared to CTL group which exhibited a basal DHA content of 3.9%. SFA (mainly stearic acid, 18:0) increased with time in all the groups, mostly at the expense of PUFA, including DHA in the DHA group and both DPA and DHA in the ALA group. The time effect on SFA was significantly more pronounced in the CTL group (+ 21% between 2 and 6 months) than in the DHA and ALA groups (+4% and +5%, respectively). Both n-6/n-3 and P/S ratios increased significantly in the CTL group while they remained unchanged in both DHA and ALA groups.

**Table 4:** Sarcolemmal membrane fatty acid profiles (% of total fatty acids) from rats fed experimental diets for 2 or 6 months.

Duration (months)	2	2	2	6	6	6	
Dietary groups	CTL	DHA	ALA	CTL	DHA	ALA	

Fatty Acids							
16:0	10.3 ± 0.28^b^	13.3 ± 0.39^a^	12.5 ± 0.36^a^	11.4 ± 0.87	12.9 ± 0.53	12.5 ± 0.38	-
18:0	21.6 ± 0.36	23.4 ± 0.36	22.8 ± 0.50	27.0 ± 1.50***	25.1 ± 0.93	24.5 ± 1.00	-
18:1 n-9	4.8 ± 0.04	4.5 ± 0.28	5.5 ± 0.12	5.0 ± 0.35^a^	4.1 ± 0.24^b^	5.2 ± 0.35^a^	-
18:1 n-7	2.9 ± 0.07	2.5 ± 0.05	2.7 ± 0.10	2.9 ± 0.17^a^	2.4 ± 0.06^b^	2.9 ± 0.13^a^	-
18:2 n-6	26.5 ± 0.75^a^	21.5 ± 0.61^b^	26.3 ± 0.60^a^	22.7 ± 1.75^b^	22.2 ± 0.87^b^	26.5 ± 1.49^a^	-
18:3 n-3	<0.1^b^	<0.1^b^	1.3 ± 0.04^a^	<0.1^b^	<0.1^b^	1.1 ± 0.12^a ^**	-
20:4 n-6	22.2 ± 0.22^a^	10.8 ± 0.24^c^	14.0 ± 0.35^b^	21.3 ± 1.11^a^	10.8 ± 0.52^c^	13.0 ± 0.30^b^	-
20:5 n-3	<0.1^c^	0.8 ± 0.04^b^	1.2 ± 0.07^a^	<0.1^b^	1.2 ± 0.25^a^	1.3 ± 0.07^a^	-
22:4 n-6	1.2 ± 0.04^a^	<0.1^b^	<0.1^b^	1.0 ± 0.05^a ^***	<0.1^b^	<0.1^b^	-
22:5 n-6	2.7 ± 0.15^a^	0.7 ± 0.02^b^	<0.1^c^	2.5 ± 0.20^a^	0.5 ± 0.02^b^	<0.1^c^	-
22:5 n-3	0.7 ± 0.03^b^	0.7 ± 0.04^b^	3.4 ± 0.15^a^	0.5 ± 0.04^b^	0.7 ± 0.13^b^	2.8 ± 0.11^a ^**	$
22:6 n-3	3.9 ± 0.10^c^	19.2 ± 1.01^a^	7.5 ± 0.32^b^	2.7 ± 0.10^c ^*	17.3 ± 1.00^a ^*	6.5 ± 0.47^b ^*	-

SFA	32.6 ± 0.28	37.5 ± 0.58	36.1 ± 0.60	39.5 ± 2.43**	38.8 ± 1.45	37.9 ± 1.35	-
MUFA	8.8 ± 0.11^a^	7.7 ± 0.34^b^	9.1 ± 0.25^a^	9.1 ± 0.50^a^	7.8 ± 0.32^b^	10.0 ± 0.47^a^	-
PUFA	58.7 ± 0.29	54.8 ± 0.83	54.8 ± 0.59	51.4 ± 2.90**	53.4 ± 1.74	52.1 ± 1.45	-
n-6 PUFA	53.7 ± 0.29^a^	33.8 ± 0.61^c^	41.2 ± 0.39^b^	48.0 ± 2.78^a ^*	34.0 ± 1.28^c^	40.1 ± 1.29^b^	-
n-3 PUFA	4.9 ± 0.25^c^	20.9 ± 1.00^a^	13.6 ± 0.28^b^	3.3 ± 0.15^c ^*	19.4 ± 0.88^a^	12.0 ± 0.62^b^	-
LA/AA ratio	1.2 ± 0.04^b^	2.0 ± 0.06^a^	1.9 ± 0.08^a^	1.1 ± 0.04^b^	2.1 ± 0.07^a^	2.0 ± 0.14^a^	-
AA/DHA ratio	5.7 ± 0.15^a^	0.6 ± 0.03^c^	1.9 ± 0.10^b^	8.0 ± 0.17^a ^***	0.6 ± 0.04^c^	2.1 ± 0.16^b^	-
n-6/n-3 ratio	11.0 ± 0.57^a^	1.6 ± 0.10^c^	3.0 ± 0.05^b^	14.6 ± 0.50^a ^***	1.8 ± 0.10^c^	3.4 ± 0.21^b^	-
P/S ratio	1.8 ± 0.02	1.5 ± 0.04	1.5 ± 0.04	0.8 ± 0.09^b ^**	1.4 ± 0.09^a^	1.4 ± 0.08^a^	-

SFA. MUFA. and PUFA. saturated. monounsaturated. and polyunsaturated FA; CTL. n-3 PUFA free diet; DHA. docosahexaenoic acid-rich diet;ALA. alpha linolenic acid-rich diet.

Data are means ± SEM. n = 6. Dietary effect (CTL versus DHA versus ALA) was compared in rats from the same dietary treatment duration and values with different letters are statistically different. a>b>c. p < 0.01. Time effect (2 months versus 6 months) was compared between rats from the same dietary group. with * = p < 0.05; ** = p < 0.01. *** = p < 0.001). Symbol $ denoted significant cross-interaction. p < 0.05.

#### NU fraction

Among the various fractions, NU exhibited the lowest PUFA content (≤ content 50%) and the highest MUFA content (12–14%) (Table 5). In all groups, palmitic acid (16:0) and oleic acid (18:1 n-9, OA) were predominantly incorporated in the NU fraction. NU fraction exhibited a 2-fold higher level of OA than the other membranes in the CTL group after 2 months of diet (10% of total fatty acids). The NU fatty acid profile was very influenced by the dietary fatty acids as well. Feeding the rats an n-3 PUFA diet resulted in a NU DHA content higher in the DHA and ALA groups (>20% and 7–8%, respectively). Interestingly, in the ALA group, the incorporation of ALA (2.5%) and DPA (4%) was higher in the nuclear membrane than in any other membrane fraction. As compared to the other membrane fractions, in the two n-3 PUFA-fed groups, NU displayed a higher incorporation of n-3 PUFA and the lowest n-6/n-3 ratio. Only the 18:1 n-7 (end-product of the delta 9 desaturase effect on stearic acid) appeared significantly affected in the CTL group with time. The experimental duration affected three parameters (total SFA, P/S and AA/DHA ratios), but the time effect remained extremely weak in NU. Only the CTL group displayed a significant increase in the AA/DHA ratio with time (significant diet/duration cross-interaction) while this ratio remained unchanged in the two n-3 PUFA groups.

**Table 5:** Nuclear membrane fatty acid profiles (% of total fatty acids) from rats fed experimental diets for 2 or 6 months.

Duration (months)	2	2	2	6	6	6	
Dietary groups	CTL	DHA	ALA	CTL	DHA	ALA	

Fatty Acids							CI
16:0	13.9 ± 0.4^b^	15.6 ± 0.46^a^	14.6 ± 0.33^ab^	13.2 ± 0.27^b^	15.3 ± 0.30^a^	14.7 ± 0.29^ab^	-
18:0	21.0 ± 0.37	20.3 ± 0.55	20.9 ± 0.47	21.7 ± 0.25	22.0 ± 0.54	21.7 ± 0.24	-
18:1 n-9	9.7 ± 0.68^a^	8.2 ± 0.46^b^	8.7 ± 0.34^ab^	9.5 ± 0.61^a^	8.2 ± 0.57^b^	9.2 ± 0.36^ab^	-
18:1 n-7	3.0 ± 0.08^a^	2.3 ± 0.07^b^	2.7 ± 0.10^b^	2.7 ± 0.03^a ^*	2.2 ± 0.07^b^	2.8 ± 0.05^b^	-
18:2 n-6	23.4 ± 0.65^a^	16.7 ± 0.53^c^	21.2 ± 0.92^b^	22.8 ± 0.48^a^	16.9 ± 0.28^c^	20.5 ± 0.51^b^	-
18:3 n-3	<0.1^b^	<0.1^b^	2.5 ± 0.09^a^	<0.1^b^	<0.1^b^	2.7 ± 0.12^a^	-
20:4 n-6	18.0 ± 0.82^a^	9.4 ± 0.28^c^	13.1 ± 0.55^b^	19.6 ± 0.72^a^	9.5 ± 0.59^c^	12.3 ± 0.55^b^	-
20:5 n-3	<0.1^c^	0.8 ± 0.05^b^	1.2 ± 0.06^a^	<0.1^c^	0.9 ± 0.02^b^	1.3 ± 0.05^a^	-
22:4 n-6	1.3 ± 0.08^a^	<0.1^b^	<0.1^b^	1.3 ± 0.07^a^	<0.1^b^	<0.1^b^	
22:5 n-6	2.4 ± 0.11^a^	0.9 ± 0.02^b^	<0.1^c^	2.6 ± 0.28^a^	0.8 ± 0.0^b^	<0.1^c^	
22:5 n-3	0.6 ± 0.07^c^	1.0 ± 0.07^b^	3.8 ± 0.26^a^	0.6 ± 0.05^c^	0.9 ± 0.03^b^	3.8 ± 0.05^a^	-
22:6 n-3	3.0 ± 0.34^c^	21.2 ± 0.55^a^	7.8 ± 0.34^b^	2.7 ± 0.22^c^	20.0 ± 0.33^a ^*	7.4 ± 0.41^b^	-

SFA	36.2 ± 0.36	37.3 ± 0.41	36.5 ± 0.39	36.4 ± 0.34^b^	38.7 ± 0.34^a ^**	37.7 ± 0.18**	-
MUFA	14.2 ± 0.66	11.9 ± 0.64	13.0 ± 0.50	13.3 ± 0.66	11.7 ± 0.69	13.5 ± 0.38	-
PUFA	49.6 ± 0.86	50.8 ± 0.67	50.6 ± 0.56	50.3 ± 0.85	49.7 ± 0.48	48.8 ± 0.45	-
n-6 PUFA	45.8 ± 0.68^a^	27.6 ± 0.68^c^	35.3 ± 0.84^b^	46.8 ± 0.61^a^	27.7 ± 0.58^c^	33.5 ± 0.58^b^	-
n-3 PUFA	3.8 ± 0.39^c^	23.2 ± 0.63^a^	15.3 ± 0.58^b^	3.4 ± 0.25^c^	22.0 ± 0.30^a^	15.3 ± 0.41^b^	-
LA/AA ratio	1.3 ± 0.09^b^	1.8 ± 0.07^a^	1.6 ± 0.11^ab^	1.2 ± 0.07^b^	1.8 ± 0.12^a^	1.7 ± 0.10^a^	-
AA/DHA ratio	6.1 ± 0.48^a^	0.5 ± 0.02^c^	1.7 ± 0.09^b^	7.4 ± 0.36^a ^*	0.5 ± 0.03^c^	1.7 ± 0.12^b^	$
n-6/n-3 ratio	12.5 ± 1.41^a^	1.2 ± 0.05^c^	2.3 ± 0.15^b^	14.0 ± 0.89^a^	1.3 ± 0.04^c^	2.2 ± 0.09^b^	-
P/S ratio	1.4 ± 0.03	1.4 ± 0.03	1.4 ± 0.03	1.4 ± 0.03	1.3 ± 0.01*	1.3 ± 0.02*	-

SFA. MUFA. and PUFA. saturated. monounsaturated. and polyunsaturated FA; CTL. n-3 PUFA free diet; DHA. docosahexaenoic acid-rich diet; ALA. alpha linolenic acid-rich diet.

Data are means ± SEM. n = 6. Dietary effect (CTL versus DHA versus ALA) was compared in rats from the same dietary treatment duration and values with different letters are statistically different. a>b>c. p < 0.01. Time effect (2 months versus 6 months) was compared between rats from the same dietary group. with * = p < 0.05; ** = p < 0.01. *** = p < 0.001). Symbol $ denoted significant cross-interaction. p < 0.05.

#### MI fraction

The MI fraction exhibited after 2 months of diet a highly unsaturated fatty acid profile with a total PUFA content close to 55–58%, and a SFA and MUFA contents about 33–36% and 7–10%, respectively (Table 6). All the fatty acids were influenced by the diet, including those which dietary supply was similar in the 3 groups. The dietary-induced changes in MI fatty acid profile were roughly similar to those observed in the others membrane fractions. Both ALA and DHA diets induced a strong increase in n-3 PUFA content (22–26% and 14–16% in the DHA group and the ALA group, respectively, as compared to 4–5% in CTL group) and a corresponding decrease in n-6 PUFA content particularly in the DHA group (from 55% in CTL group to 34% and 43% in DHA and ALA groups, respectively after 6 months). A time effect was observed for several individual fatty acids in all the groups. Whatever the group, the 18:1 n-7 decreased while 18:2 n-6 increased (a major component of cardiolipins); 18:0 decreased in the DHA group and 18:1 n-9 decreased in the two n-3 PUFA-fed groups (DHA and ALA). DHA (and DPA) decreased significantly in the CTL and DHA groups but not in the ALA group. The increase in LA content was associated with a significant decrease in SFA in the DHA group only. In the CTL group only, the n-6/n-3 ratio, the P/S ratio and the AA/DHA ratio increased significantly with time as a consequence of the n-6 PUFA rise, SFA increase and the tendency of AA to increase and DHA to decrease (significant diet/duration cross-interaction), respectively. In the DHA and ALA groups only, the LA/AA ratio increased with time (significant diet/duration cross-interaction), while remaining constant in the CTL group.

**Table 6:** Mitochondrial fatty acid profiles (% of total fatty acids) from rats fed experimental diets for 2 or 6 months.

Duration (months)	2	2	2	6	6	6	
Dietary groups	CTL	DHA	ALA	CTL	DHA	ALA	

Fatty acids							CI
16:0	10.5 ± 0.37^b^	12.5 ± 0.51^a^	11.9 ± 0.13^a^	10.2 ± 0.33	11 ± 1.14**	10.9 ± 0.07	-
18:0	23.2 ± 0.29	22.3 ± 0.32	22.8 ± 0.12	21.4 ± 0.32^ab^	21 ± 2.22^b ^*	22.5 ± 0.14^a^	-
18:1 n-9	5.4 ± 0.51	5.2 ± 0.35	6 ± 0.18	4.8 ± 0.11	3.8 ± 0.46*	4.7 ± 0.13*	-
18:1 n-7	3 ± 0.06^a^	2.3 ± 0.05^b^	2.8 ± 0.05^c^	2.4 ± 0.03^a ^***	1.9 ± 0.16^b ^***	2.6 ± 0.06^a ^***	-
18:2 n-6	22.7 ± 0.66^b^	17.4 ± 0.94^c^	23 ± 0.32^a^	25.4 ± 0.59^b ^***	21.2 ± 2.19^c ^***	27.6 ± 0.53^a ^***	-
18:3 n-3	<0.01^b^	<0.01^b^	1.6 ± 0.06^a^	<0.01^b^	<0.01^b^	1.3 ± 0.04^a ^***	-
20:4 n-6	23.1 ± 0.73^a^	11.1 ± 0.26^c^	14.8 ± 0.37^b^	24.8 ± 0.34^a ^*	11.6 ± 1.45^b^	14.2 ± 0.48^c^	-
20:5 n-3	<0.01	0.9 ± 0.04	1.3 ± 0.07	<0.01	1.0 ± 0.12^b^	1.4 ± 0.05^a^	-
22:4 n-6	1.4 ± 0.05^a^	<0.01^b^	<0.01^b^	1.3 ± 0.04^a^	<0.01^b^	<0.01^b^	-
22:5 n-6	3.2 ± 0.24^a^	0.7 ± 0.14^b^	<0.01^c^	2.9 ± 0.23^a^	0.7 ± 0.09^b^	<0.01^c^	-
22:5 n-3	0.7 ± 0.03^b^	0.9 ± 0.05^b^	3.7 ± 0.08^a^	0.7 ± 0.02^b^	0.7 ± 0.09^b ^***	3.6 ± 0.06^a^	-
22:6 n-3	4.4 ± 0.28^c^	24.5 ± 0.97^a^	9.4 ± 0.25^b^	3.4 ± 0.10^c ^***	20.4 ± 2.73^a ^***	8.2 ± 0.35^b^	-

SFA	34.4 ± 0.21	35.5 ± 0.36	35.4 ± 0.14	32.5 ± 0.46	32.9 ± 1.02**	34.6 ± 0.12	-
MUFA	9.3 ± 0.6	8.4 ± 0.46	9.8 ± 0.26	8.4 ± 0.20^a^	6.7 ± 0.45^b ^***	8.4 ± 0.22^a^	-
PUFA	56.3 ± 0.74	56.1 ± 0.72	54.8 ± 0.37	59.1 ± 0.63	56.2 ± 2.06	57.0 ± 0.22	-
n-6 PUFA	51 ± 0.52^a^	29.7 ± 1.19^b^	38.7 ± 0.19^c^	54.8 ± 0.66^a ^***	33.9 ± 1.04^b ^***	42.5 ± 0.42^c ^***	-
n-3 PUFA	5.2 ± 0.30^c^	26.4 ± 1.01^a^	16.1 ± 0.30^b^	4.2 ± 0.11^c^	22.3 ± 1.39^a ^***	14.8 ± 0.36^b^	-
LA/AA	1.0 ± 0.06^b^	1.6 ± 0.07^a^	1.6 ± 0.06^a^	1.0 ± 0.02^b^	1.8 ± 0.06^a ^**	2.0 ± 0.09^a ^**	$
AA/DHA	5.3 ± 0.22^a^	0.5 ± 0.03^c^	1.6 ± 0.04^b^	7.4 ± 0.27^a ^***	0.6 ± 0.04^c^	1.8 ± 0.11^b^	$
n-6/n-3 ratio	9.9 ± 0.48^a^	1.1 ± 0.09^c^	2.4 ± 0.05^b^	13.1 ± 0.43^a ^***	1.5 ± 0.09^c^	2.9 ± 0.10^b^	$
P/S ratio	1.6 ± 0.03	1.6 ± 0.04	1.6 ± 0.02	1.8 ± 0.05^a ^***	1.7 ± 0.02^a^	0.6 ± 0.01^b^	$

SFA. MUFA. and PUFA. saturated. monounsaturated. and polyunsaturated FA; CTL. n-3 PUFA free diet; DHA. docosahexaenoic acid-rich diet; ALA. alpha linolenic acid-rich diet.

Data are means ± SEM. n = 6. Dietary effect (CTL versus DHA versus ALA) was compared in rats from the same dietary treatment duration and values with different letters are statistically different. a>b>c. p < 0.01. Time effect (2 months versus 6 months) was compared between rats from the same dietary group. with * = p < 0.05; ** = p < 0.01. *** = p < 0.001). Symbol $ denoted significant cross-interaction. p < 0.05.

#### SR fraction

The SR also exhibited after a 2-month dietary period a highly unsaturated fatty acid profile whatever the dietary group (58–59% of PUFA and 6–8% of MUFA) and the lowest SFA content (32–34%) as compared to the other membrane fractions (Table 7). Like the other membranes, the SR fatty acid profile was affected by the dietary fatty acids. In the DHA group, the DHA content after 2 months (23%) was higher than in the other membrane fractions. In the ALA group, SR also displayed the highest DHA content (although much lower than in the DHA group). Thereafter, the DHA content in SR decreased significantly with time in the CTL and DHA groups, but remained unchanged in the ALA group. Similarly the total n-3 PUFA decreased significantly with time in the DHA and CTL groups, but kept increasing in the ALA group that exhibited a slight increase in DHA and a higher increase in both EPA and DPA. As a consequence, the n-6/n-3 ratio significantly increased between 2 and 6 months in the CTL group and tended to increase in the DHA group, but remained unchanged in the ALA group. The individual n-3 fatty acid changes were more pronounced in the DHA group than in the ALA group that, exhibiting different time-induced changes, carried several significant diet/duration cross-interaction (n-3 PUFA, AA/DHA). Interestingly none of the total SFA, MUFA or PUFA was affected by the duration of the experiment, reflecting a relative conservation of the SR fatty acid profile with time.

**Table 7:** Sarcoplasmic reticulum fatty acid profiles (% of total fatty acids) from rats fed experimental diets for 2 or 6 months.

Duration (months)	2	2	2	6	6	6	
Dietary groups	CTL	DHA	ALA	CTL	DHA	ALA	

Fatty acids							CI
16:0	9.1 ± 0.36^c^	11.2 ± 0.35^a^	10.5 ± 0.33^b^	9.5 ± 0.46^b^	11 ± 0.22^a^	10.2 ± 0.05^ab^	-
18:0	22.9 ± 0.31	22.5 ± 0.36	22.3 ± 0.26	23.2 ± 0.4	22.6 ± 0.22	22.8 ± 0.12	-
18:1 n-9	4.6 ± 0.18^a^	3.7 ± 0.13^b^	4.9 ± 0.10^a^	4.1 ± 0.13^a ^**	3.4 ± 0.14^b^	4.1 ± 0.1^a ^**	-
18:1 n-7	2.9 ± 0.06^a^	2.3 ± 0.05^c^	2.6 ± 0.07^b^	2.5 ± 0.08^a ^***	2.1 ± 0.07^b ^***	2.6 ± 0.05^a^	$
18:2 n-6	25.8 ± 0.54^b^	20.9 ± 0.76^c^	26.9 ± 0.61^a^	26.1 ± 0.45^a^	23.4 ± 0.36^b ^*	26.8 ± 0.65^a^	-
18:3 n-3	<0.01	<0.01	1.3 ± 0.05	<0.01	<0.01	1.2 ± 0.04**	
20:4 n-6	23.3 ± 0.57^a^	11.7 ± 0.27^b^	14.8 ± 0.41^b^	24.4 ± 0.64^a^	11.8 ± 0.51^b^	15.1 ± 0.58^b^	-
20:5 n-3	<0.01	0.9 ± 0.04	1.1 ± 0.28	<0.01	1.0 ± 0.03^b^	1.5 ± 0.03^a^*	
22:4 n-6	1.3 ± 0.03	<0.01	<0.01	1.1 ± 0.04***	<0.01	<0.01	
22:5 n-6	3.1 ± 0.23^a^	0.8 ± 0.02^a^	<0.01	2.8 ± 0.24^b^	0.7 ± 0.01^a^	<0.01	
22:5 n-3	0.7 ± 0.03^b^	0.8 ± 0.04^b^	3.5 ± 0.13^a^	0.6 ± 0.03^b^	0.7 ± 0.03^b^	3.9 ± 0.06^a ^*	$
22:6 n-3	4.2 ± 0.16^c^	23 ± 0.70^a^	8.9 ± 0.46^b^	3.2 ± 0.14^c^	20.9 ± 0.24^a ^**	9 ± 0.36^b^	$

SFA	32.8 ± 0.41	34.4 ± 0.32	33.8 ± 0.33	33.2 ± 0.84	34.2 ± 0.12	33.6 ± 0.09	-
MUFA	8.2 ± 0.24^a^	6.9 ± 0.16^b^	8.6 ± 0.28^a^	7.8 ± 0.30^a^	6.6 ± 0.23^b^	8 ± 0.08^a^	-
PUFA	59 ± 0.63	58.7 ± 0.38	57.6 ± 0.52	58.9 ± 1.11	59.1 ± 0.26	58.4 ± 0.09	-
n-6 PUFA	54 ± 0.65^a^	34 ± 0.79^c^	42.7 ± 0.66^b^	54.9 ± 1.01^a^	36.4 ± 0.43^c ^*	42.6 ± 0.35^b^	-
n-3 PUFA	5 ± 0.17^c^	24.8 ± 0.70^a^	14.8 ± 0.68^b^	4 ± 0.16^c ^*	22.7 ± 0.24^a ^*	15.7 ± 0.36^b^	$
LA/AA	1.1 ± 0.05^b^	1.8 ± 0.08^a^	1.8 ± 0.07^a^	1.1 ± 0.02^b^	2 ± 0.10^a^	1.8 ± 0.10^a^	-
AA/DHA	5.6 ± 0.20^a^	0.5 ± 0.02^c^	1.7 ± 0.09^b^	7.7 ± 0.26^a ^***	0.6 ± 0.03^c^	1.7 ± 0.10^b^	$
n-6/n-3 ratio	10.9 ± 0.43^a^	1.4 ± 0.07^c^	2.9 ± 0.18^b^	13.9 ± 0.41^a ^***	1.6 ± 0.03^c^	2.7 ± 0.09^b^	$
P/S ratio	1.8 ± 0.04	1.7 ± 0.03	1.7 ± 0.03	1.7 ± 0.07	1.7 ± 0.01	1.7 ± 0.01	-

SFA. MUFA. and PUFA. saturated. monounsaturated. and polyunsaturated FA; CTL. n-3 PUFA free diet; DHA. docosahexaenoic acid-rich diet; ALA. alpha linolenic acid-rich diet.

Data are means ± SEM. n = 6. Dietary effect (CTL versus DHA versus ALA) was compared in rats from the same dietary treatment duration and values with different letters are statistically different. a>b>c. p < 0.01. Time effect (2 months versus 6 months) was compared between rats from the same dietary group. with * = p < 0.05; ** = p < 0.01. *** = p < 0.001). Symbol $ denoted significant cross-interaction. p < 0.05.

#### Unsaturation index

**Figure 2:**
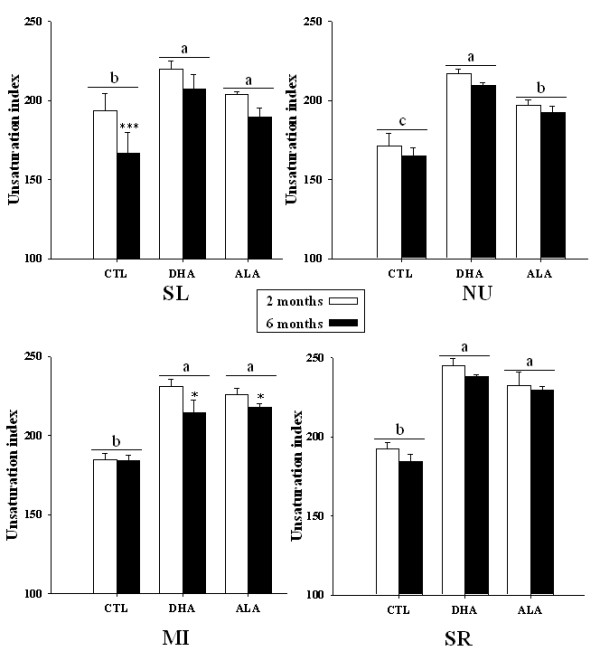
**Membrane unsaturation indexes**. The unsaturation index is the sum of the percentages of each FA in a fraction multiplying by the number of the considered fatty acid double bound. SL, sarcolemmal; NU, nuclear; MI, mitochondrial; SR, sarcoplasmic membrane. Data are means ± SEM, n = 6. Dietary effect (CTL versus DHA versus ALA) was compared in rats from the same dietary treatment duration and values with different letters are statistically different, a>b>c, p < 0.01. Time effect (2 months versus 6 months) was compared between rats from the same dietary group, with * = p < 0.05 and *** = p < 0.001.

The effects of dietary fatty acids and duration on the Unsaturation Index (UI) were roughly similar in all the subcellular membrane fractions (Figure 2). In each fraction, the UI was significantly lower in the CTL group than in the two n-3 PUFA-fed groups (p < 0.001). Among the fractions, the NU membrane was the most saturated in the CTL group after 2 and 6 months of dietary treatment (UI = 171 and 165, respectively). Feeding the rats a DHA or ALA diet increased significantly the UI in NU, which remained unaffected by duration. NU displayed a more unsaturated profile in the DHA group (UI = 217 and 210 after 2 and 6 months) than in ALA group (UI = 197 and 192 after 2 and 6 months) (p < 0.001). On the contrary, SR appeared as the most unsaturated fraction in the DHA group (UI = 244 and 238 after 2 and 6 months, respectively) and ALA group (UI = 232 and 229 after 2 and 6 months, respectively). In SL fraction, a significant decreased with time was observed in the CTL group (UI = 193 and 166 after 2 and 6 months, respectively, p < 0.001). Interestingly, this decrease was prevented with the two n-3 diets, ALA and DHA (that exhibited no difference of UI between 2 and 6 months). Conversely, although the UI in MI was not influenced by duration in the CTL group, it was moderately reduced with time in the ALA group (UI = 226 and 217 after 2 and 6 months, respectively, significant different with p < 0.001) and DHA groups (UI = 230 and 214 after 2 and 6 months, respectively, significant different with p < 0.001). In the SL, SR and MI fractions, the UI was similar in the ALA and DHA groups (and different from CTL group, p < 0.001) both after 2 months and after 6 months.

## Discussion

The aim of this study was to determine the effect of a long-term ALA *vs *DHA intake subcellular membrane composition that may explain the delay required (6 months, vs 2 months, respectively) to obtain a similar functional cardiac response (basal heart and chronotropic response) [8]. Based on our previous observations, we hypothesized that the fatty acid composition of the cardiac subcellular membranes, particularly the SR and SL, because of their strong involvement in antiarrhythmic effects [20] and cardiac excitation-contraction coupling [21] could be differently affected by both dietary supply and duration.

### Whole heart

The whole cardiac phospholipid fatty acid content was rich in PUFA (± 56%), in the CTL group mostly from the n-6 PUFA series (52%), but also from n-3 PUFA (± 5%, mainly DHA). This low DHA content, which may represent a minimum cardiac requirement, should be inherited, linked to the maternal stores or dietary habits [22], or most probably in our case, due to the standard diet (containing n-3 PUFA) used to maintain these rats from weaning to young adult age, prior the beginning of the present experiment. DHA intake is recommended to increase the DHA content in cardiac phospholipids [23,24]. We and others reported that a DHA-rich diet is able to increase the cardiac DHA content up to 18–20% of total fatty acids [13,24,25]. In our experimental conditions in rats, ALA intake allowed cardiac membrane DHA to increase up to 10% *vs*. 24% with a DHA-rich diet, as previously reported [8].

Most of ALA is stored in adipose tissue, and some other organs [26]. The carbon recycling pathway for the *de novo *lipid synthesis consumes the majority of ALA and LA (rather than palmitate), although another part is used in beta-oxidation, and a small part converted to long-chain PUFAs [27]. ALA can be efficiently elongated to DHA in numerous mammalian tissues, including liver and brain [9,28]. The rat cardiomyocyte was shown to be unable to produce DHA from its precursors [29] and this was recently confirmed *in vivo *[28]. The cardiac DHA content in the ALA group may thus be provided from plasma, after transformation of ALA in the liver, as suggested in the literature [28]. It is usually assumed that LA lowers EPA synthesis because LA is a competitive inhibitor of the metabolism of ALA since the 2 pathways use the same enzymes [6,30]. In this study ALA was significantly converted into higher n-3 metabolites, most probably because ALA replaced a significant part of LA in the diet. Moreover we observed that the stepwise increase in DHA was paralleled by a stepwise decrease in AA, as previously reported [8,11,13,25]. Besides the dietary-induced changes, we observed that the changes which occurred with time in plasma fatty acid profiles affected the membrane fatty acid profiles. The n-6/n-3 ratio increased with time in the whole heart membrane and in several subcellular membranes including SL, SR and MI but only in the CTL-fed rats. Moreover, the decrease with duration in plasma P/S ratio affected the P/S ratio only in the SL fraction and only in the CTL group.

### Cardiac NU fraction

The n-3 PUFA content of the NU membrane was in the same range as the other subcellular membranes, with a particular incorporation of ALA and DPA in the ALA group. PUFA (and mainly DHA) were reported to bind to retinoid-X-receptor (RXR) [31], nuclear receptors that play a major role in gene and protein expression involved in lipid transport, biosynthesis and metabolism. Furthermore n-3 PUFA can also bind and stimulate PPARs that act as lipid biosensors [32]. The high n-3 PUFA level observed in the NU membrane is of interest and, because of a relative proximity, may contribute to their effects on gene regulation and expression. With time the NU membrane with its higher MUFA content among the subcellular membranes, remained rather stable.

### Cardiac MI fraction

The MI membrane was characterized by a significant increase of the AA/DHA and n-6/n-3 ratios with time in the CTL-fed rats. This phenomenon was reported in the whole heart and particularly in mitochondria as a consequence of ageing [23,33]. In this study, we observed such alterations attributed to a loss of long chain n-3 PUFA in MI membrane early in lifespan, in 8 months-old rats. These time-related loss in MI long chain n-3 PUFA was prevented by an n-3 PUFA dietary supply, according to the present results and the literature [23,33]. Although decreasing with time, the MI DHA content remained high after 6 months in the rats fed a DHA-rich diet. Interestingly, in the rats fed an ALA-rich flour based diet, the age-related loss in DHA was not significant. Such changes in MI fatty acid profile may have profound functional consequences on the efficiency of membrane proteins involved in numerous processes including ion homeostasis and intracellular signal transduction, free radical metabolism and energy production [33]. In the diabetic heart, the functional activity of mitochondria is reduced partially because of the alteration of cardiolipin turnover, which can be improved when DHA increased in the mitochondrial membrane [34]. The effect of DHA on PPARα may contribute to the stimulation of cardiolipin synthesis, as already suggested [35]. In both DHA and ALA groups, the LA/AA ratio increased with time in MI, but not in the CTL group. As far as cardiolipin contains approximately 75% of LA, a stimulation of cardiolipin synthesis by n-3 long chain PUFA may account increased content of LA in MI fraction. Several authors suggested that the loss of n-3 PUFA with time may constitute a protection against lipid peroxidation [36], although recent works reported that restoring a high level of n-3 PUFA in old rats reversed cellular dysfunctions, including mitochondrial energy production, tolerance to ischemia and reperfusion and Ca^2+ ^metabolism [33].

### Cardiac SL and SR fractions

The most important time-induced variations occurred in the SL in the CTL group. The SFA content increased while the total PUFA significantly decreased affecting significantly n-6 and even more n-3 PUFA contents, as revealed by the highly significant increase in n-6/n-3 and AA/DHA ratios. Like MI, SL displayed a significant decrease in DHA with time, whatever the dietary group and the dietary n-3 PUFA supply strongly attenuated the time-induced total n-3 PUFA loss in SL. To our knowledge, the prevention by dietary n-3 PUFA of the time-induced changes in cardiac SL has not been reported in the literature.

Among the different membrane fractions investigated, the SR was the richest in PUFA and this high PUFA content was preserved throughout the experimental duration, whatever the dietary group. Unlike other membranes, SR displayed a significant cross-interaction between duration and diets, in the two n-3 PUFA groups. The SR DHA content decreased in DHA-fed rats whereas it was maintained and DPA was increased in ALA-fed rats. As a consequence, The total C22 n-3 content in SR slightly decreased with time in the DHA group but kept increasing with time in the ALA group.

These modifications in SL and SR may affect the activity of ion channels and proteins, mainly those involved in the excitation-contraction coupling. Dietary n-3 PUFA-induced n-3 PUFA membrane enrichment was reported to play a key role in cardiac β-adrenergic function [8,37] and calcium signalling [20,21,38]. Numerous studies, mostly in cultured cardiac cells, strongly suggested that changes of the physical state of the SL membrane, particularly affecting the phospholipid fatty acids of the membrane micro-domain surrounding the receptors, influence or co-regulate the functionality and the number of assayable of β-adrenergic receptors [39,40]. Other functions were reported to be influenced by membrane n-3 PUFA, including transient outward current, L-type Ca^2+ ^current, voltage-dependant Na^+ ^current, and Ca^2+^-Mg^2+^-ATPase in SL and ryanodine channel, Na^+^-Ca^2+^-exchanger, Ca^2+ ^uptake in SR (see for review [41]).

Moreover, the age-related changes in the activity of these various membrane-bound protein systems were also shown to be prevented by long chain n-3 PUFA supply (see for review [42]). The ALA-rich diet did not allow to reach the same DHA content than the DHA diet. However in the ALA group, the total n-3 PUFA in SR kept increasing with time, while it decreased in the other groups. By comparison with the functional effects previously reported with the same diets [8], the present study suggests that the delayed effect of ALA diet (on heart rate and adrenergic response) may be due to the evolution with time of individual subcellular membranes: (i) in SR, the C22 n-3 content kept increasing after 6 months of diet with ALA supply but not with DHA supply, suggesting the importance of C22 n-3 in the regulation of ryanodine receptor function; (ii) in SL, although decreasing with time, the DHA (and DPA) content remains high enough to account for the regulation of beta-adrenergic receptor function both in ALA group and DHA group. Whatever the membrane fraction considered, DHA and ALA induced a significant rise in membrane unsaturation after 2 months, suggesting an increased membrane fluidity. Recently [43] the introduction of DHA-rich domains into the sarcolemma and their coexistence with lipid rafts was suggested to account for the diversity of health benefits associated to DHA intake. The changes in the conformation of signalling-involved proteins, when they move among these disparate domains, may have the potential to modulate cell function.

## Conclusion

Although not achieving a similar level of DHA in the membranes, an ALA-rich diet was characterized by an ability to keep increasing the total n-3 PUFA during the time course of the experiment, mainly in SR. This unique property may explain partly why in previous paper, the adrenergic response was observed after 2 months with a DHA diet, but after 6 months with an ALA diet. The difference of n-3 PUFA content in plasma membrane (SL) and sarcoplasmic reticulum (SR), both involved in the chronotropic response to adrenergic stimulation, decreased with time between in ALA group and DHA group. Indeed the n-3 PUFA content decreased in the DHA group after 6 months and increased after 2 months in the ALA group. Although the n-3 PUFA supply (either ALA or DHA) prevented the alteration of membrane P/S ratio, whatever the membrane fraction and whatever the diet duration. These observations brought some evidences of the interest of introducing as well in longer-term dietary experiments, the essential fatty acid ALA as compared to the end-product metabolite in the n-3 pathway, namely DHA, in order to investigate a possible dietary impact on health.

## List of abbreviations used

AA: arachidonic acid, 20:4 n-6; ALA: linolenic acid, 18:3 n-3; DHA: docosahexaenoic acid, 22:6 n-3; DPA: docosapentaenoic acid, 22:5 n-3; EPA: eicosapentaenoic acid, 20:5 n-3; LA: linoleic acid, 18:2 n-6; MUFA: monounsaturated fatty acid; OA: oleic acid, 18:1 n-9; P/S: polyunsaturated fatty acid/saturated fatty acid ratio; PUFA: polyunsaturated fatty acid; SA: stearic acid, 18:0; SFA: saturated fatty acid; UI: unsaturation index

## Competing interests

The authors declare that they have no competing interests.

## Authors' contributions

AB, co-principal investigators, carried out the cardiac subcellular membrane fractioning and the Western Blot characterizing those fractions, and drafted the manuscript. MG made substantial contributions concerning the acquisition of data by carrying out the *in vivo *part of the study including rat feeding, weighting and blood sampling. JPM made substantial contributions concerning the analysis by carrying out the methyl ester fatty acid analyses by gas chromatography. PW participated in the design of the study. AG conceived and participated in the design of the study, have been involved in drafting the manuscript and revising it critically for important intellectual content. DRR, co-principal investigator, conceived and participated in the design of the study and coordination, performed the preparation of fatty acids prior analyses and the statistical analysis, drafted and revised the manuscript. All authors read and approved the final manuscript.

## Authors' information

AB (PhD student) was one main investigator of the study, amandinebrochot@gmail.com.

MG was a research engineer involved in physiology and nutritional studies (INRA), marine.guinot@jouy.inra.fr.

JPM was a research engineer involved in biochemical analyses by gas chromatography (INRA), jean-paul.macaire@jouy.inra.fr.

PW was our sponsor and provider of vegetable source of ALA (Valorex), p.weill@valorex.com.

AG (PhD, research director INRA) was the supervisor of AB's thesis and the director of the laboratory, alain.grynberg@jouy.inra.fr.

DRR (PhD, researcher senior INRA) was the principal coordinator and other main investigator of the study, delphine.rousseau@jouy.inra.fr.

## References

[B1] Palsdottir H, Hunte C (2004). Lipids in membrane protein structures. Biochim Biophys Acta.

[B2] Gebauer SK, Psota TL, Harris WS, Kris-Etherton PM (2006). n-3 fatty acid dietary recommendations and food sources to achieve essentiality and cardiovascular benefits. Am J Clin Nutr.

[B3] Holub DJ, Holub BJ (2004). Omega-3 fatty acids from fish oils and cardiovascular disease. Mol Cell Biochem.

[B4] Wang C, Harris WS, Chung M, Lichtenstein AH, Balk EM, Kupelnick B, Jordan HS, Lau J (2006). n-3 Fatty acids from fish or fish-oil supplements, but not alpha-linolenic acid, benefit cardiovascular disease outcomes in primary- and secondary-prevention studies: a systematic review. Am J Clin Nutr.

[B5] Russo GL (2008). Dietary n-6 and n-3 polyunsaturated fatty acids: From biochemistry to clinical implications in cardiovascular prevention. Biochem Pharmacol.

[B6] Burdge GC (2006). Metabolism of alpha-linolenic acid in humans. Prostaglandins Leukot Essent Fatty Acids.

[B7] Plourde M, Cunnane SC (2007). Extremely limited synthesis of long chain polyunsaturates in adults: implications for their dietary essentiality and use as supplements. Appl Physiol Nutr Metab.

[B8] Ayalew-Pervanchon A*, Rousseau D*, Moreau D, Assayag P, Weill P, Grynberg A (2007). Long-term effect of dietary {alpha}-linolenic acid or decosahexaenoic acid on incorporation of decosahexaenoic acid in membranes and its influence on rat heart in vivo. Am J Physiol Heart Circ Physiol.

[B9] Igarashi M, DeMar JC, Ma K, Chang L, Bell JM, Rapoport SI (2007). Upregulated liver conversion of alpha-linolenic acid to docosahexaenoic acid in rats on a 15 week n-3 PUFA-deficient diet. J Lipid Res.

[B10] Tahin QS, Blum M, Carafoli E (1981). The fatty acid composition of subcellular membranes of rat liver, heart, and brain: diet-induced modifications. Eur J Biochem.

[B11] Robbez MV, Lucas A, Gueugneau AM, Macaire JP, Paul JL, Grynberg A, Rousseau D (2008). Long-chain (n-3) polyunsaturated fatty acids prevent metabolic and vascular disorders in fructose-fed rats. J Nutr.

[B12] Grynberg A, Fournier A, Sergiel JP, Athias P (1996). Membrane docosahexaenoic acid vs. eicosapentaenoic acid and the beating function of the cardiomyocyte and its regulation through the adrenergic receptors. Lipids.

[B13] Rousseau D, Héliès-Toussaint C, Moreau D, Raederstorff D, Grynberg A (2003). Dietary n-3 PUFAs affect the blood pressure rise and cardiac impairments in a hyperinsulinemia rat model in vivo. Am J Physiol Heart Circ Physiol.

[B14] Dallongeville J, Yarnell J, Ducimetiere P, Arveiler D, Ferrieres J, Montaye M, Luc G, Evans A, Bingham A, Hass B, Ruidavets JB, Amouyel P (2003). Fish consumption is associated with lower heart rates. Circulation.

[B15] Austria JA, Richard MN, Chahine MN, Edel AL, Malcolmson LJ, Dupasquier CM, Pierce GN (2008). Bioavailability of alpha-linolenic acid in subjects after ingestion of three different forms of flaxseed. J Am Coll Nutr.

[B16] Paris S, Fosset M, Samuel D, Ailhaud G (1977). Chick embryo plasma membrane from cardiac muscle and cultured heart cells: isolation procedure and absence of fatty acid-activating enzymes. J Mol Cell Cardiol.

[B17] Lombet A, Lazdunski M (1984). Characterization, solubilization, affinity labeling and purification of the cardiac Na+ channel using Tityus toxin gamma. Eur J Biochem.

[B18] Folch J, Lees M, Sloane Stanley GH (1957). A simple method for the isolation and purification of total lipides from animal tissues. J Biol Chem.

[B19] Morrison WR, Smith LM (1964). Preparation of fatty acid methyl esters and dimethylacetals from lipids with boron fluoride-methanol. J Lipid Res.

[B20] Xiao YF, Sigg DC, Leaf A (2005). The antiarrhythmic effect of n-3 polyunsaturated fatty acids: modulation of cardiac ion channels as a potential mechanism. J Membr Biol.

[B21] Leifert WR, Dorian CL, Jahangiri A, McMurchie EJ (2001). Dietary fish oil prevents asynchronous contractility and alters Ca(2+) handling in adult rat cardiomyocytes. J Nutr Biochem.

[B22] Cheema KK, Dent MR, Saini HK, Aroutiounova N, Tappia PS (2005). Prenatal exposure to maternal undernutrition induces adult cardiac dysfunction. Br J Nutr.

[B23] McLennan PL, Abeywardena MY, Charnock JS (1989). The influence of age and dietary fat in an animal model of sudden cardiac death. Aust N Z J Med.

[B24] Owen AJ, Peter-Przyborowska BA, Hoy AJ, McLennan PL (2004). Dietary fish oil dose- and time-response effects on cardiac phospholipid fatty acid composition. Lipids.

[B25] Rousseau D, Helies-Toussaint C, Raederstorff D, Moreau D, Grynberg A (2001). Dietary n-3 polyunsaturated fatty acids affect the development of renovascular hypertension in rats. Mol Cell Biochem.

[B26] Lin YH, Salem N (2007). Whole body distribution of deuterated linoleic and alpha-linolenic acids and their metabolites in the rat. J Lipid Res.

[B27] Cunnane SC, Ryan MA, Nadeau CR, Bazinet RP, Musa-Veloso K, McCloy U (2003). Why is carbon from some polyunsaturates extensively recycled into lipid synthesis?. Lipids.

[B28] Igarashi M, Ma K, Chang L, Bell JM, Rapoport SI (2008). Rat heart cannot synthesize docosahexaenoic acid from circulating alpha-linolenic acid because it lacks elongase-2. J Lipid Res.

[B29] Liautaud S, Grynberg A, Mourot J, Athias P (1991). Fatty acids of hearts from rats fed linseed or sunflower oil and of cultured cardiomyocytes grown on their sera. Cardioscience.

[B30] Sprecher H (2000). Metabolism of highly unsaturated n-3 and n-6 fatty acids. Biochim Biophys Acta.

[B31] Goldstein JT, Dobrzyn A, Clagett-Dame M, Pike JW, DeLuca HF (2003). Isolation and characterization of unsaturated fatty acids as natural ligands for the retinoid-X receptor. Arch Biochem Biophys.

[B32] Chawla A, Repa JJ, Evans RM, Mangelsdorf DJ (2001). Nuclear receptors and lipid physiology: opening the X-files. Science.

[B33] Pepe S (2005). Effect of dietary polyunsaturated fatty acids on age-related changes in cardiac mitochondrial membranes. Exp Gerontol.

[B34] Ovide-Bordeaux S, Grynberg A (2004). Docosahexaenoic acid affects insulin deficiency- and insulin resistance-induced alterations in cardiac mitochondria. Am J Physiol Regul Integr Comp Physiol.

[B35] Jiang YJ, Lu B, Xu FY, Gartshore J, Taylor WA, Halayko AJ, Gonzalez FJ, Takasaki J, Choy PC, Hatch GM (2004). Stimulation of cardiac cardiolipin biosynthesis by PPARalpha activation. J Lipid Res.

[B36] Pamplona R, Portero-Otin M, Sanz A, Requena J, Barja G (2004). Modification of the longevity-related degree of fatty acid unsaturation modulates oxidative damage to proteins and mitochondrial DNA in liver and brain. Exp Gerontol.

[B37] Grynberg A, Fournier A, Sergiel JP, Athias P (1995). Effect of docosahexaenoic acid and eicosapentaenoic acid in the phospholipids of rat heart muscle cells on adrenoceptor responsiveness and mechanism. J Mol Cell Cardiol.

[B38] Rinaldi B, Di Pierro P, Vitelli MR, D'Amico M, Berrino L, Rossi F, Filippelli A (2002). Effects of docosahexaenoic acid on calcium pathway in adult rat cardiomyocytes. Life Sci.

[B39] Williams S, Meij JT, Panagia V (1995). Membrane phospholipids and adrenergic receptor function. Mol Cell Biochem.

[B40] Ponsard B, Durot I, Delerive P, Oudot F, Cordelet C, Grynberg A, Athias P (1999). Cross-influence of membrane polyunsaturated fatty acids and hypoxia-reoxygenation on alpha- and beta-adrenergic function of rat cardiomyocytes. Lipids.

[B41] Siddiqui RA, Harvey KA, Zaloga GP (2008). Modulation of enzymatic activities by n-3 polyunsaturated fatty acids to support cardiovascular health. J Nutr Biochem.

[B42] Pepe S (2007). Dietary polyunsaturated fatty acids and age-related membrane changes in the heart. Ann N Y Acad Sci.

[B43] Wassall SR, Stillwell W (2008). Docosahexaenoic acid domains: the ultimate non-raft membrane domain. Chem Phys Lipids.

