# Effects of alpha-linolenic acid *vs*. docosahexaenoic acid supply on the distribution of fatty acids among the rat cardiac subcellular membranes after a short- or long-term dietary exposure

**DOI:** 10.1186/1743-7075-6-35

**Published:** 2009-09-11

**Authors:** Amandine Brochot, Marine Guinot, Daniel Auchere, Jean-Paul Macaire, Pierre Weill, Alain Grynberg, Delphine Rousseau-Ralliard

**Affiliations:** 1 Institut National de la Recherche Agronomique (INRA)-Université Paris-Sud 11, Unité Mixte de Recherche 1154, Lipides Membranaires et Régulation Fonctionnelle du Coeur et des Vaisseaux, Institut Fédératif de Recherche 141, Faculté de Pharmacie, Châtenay-Malabry, F-92296, France; 2 Société Valorex, Combourtillé, France

## Correction

A mistake has been noted in our recently published (25 March 2009) article [1]. This error appeared in the material and methods section, and concerns the content of Table 1.

**Table 1:** Formulation and fatty acid composition of the experimental diets.

	CTL diet	DHA diet	ALA diet	Extruded linseed flour ^5^
	g/kg of diet	g/kg of diet	g/kg of diet	g/kg
**Basal mix ^1^**				
Protein				200
Soy protein isolate ^2^	170	170	147	
Glucides				110
Sucrose	220	220	216	35
Cornstarch	440	440	402	
Fibers (mucilages, ...)				171
Cellulose	20	20		80
**Minerals and other components**				44
L-Cystine	5	5	5	
Choline chloride	5	5	5	
Mineral mixture ^3^	50	50	48	
Vitamin mixture ^3^	10	10	10	
**Extruded linseed flour ^4^**			**122**	
**Lipids**				280
hydrogenated coconut oil ^5^	15.2	15	11.3	
Cocoa butter ^6^	14.4	18	25.7	
Sunflower seed oil ^7^	48	17	8.9	
Rapeseed oil ^8^	2.4	10		
n-3 LCPUFA-rich oil ^9^		20		
Humidity				80

**Fatty acid composition ^10^**	**% of total FA**	**% of total FA**	**% of total FA**	**% of total FA**
14:0	4.7	4.6	3.5	-
16:0	11.2	13.2	10.2	5.9
18:0	8.5	11.4	8.4	2.9
18:1 n-9	21.7	17.5	17.0	17.3
18:2 n-6	35.5	16.9	18.2	17.7
18:3 n-3	0.6	23.3	1.4	55.1
20:5 n-3	-	-	2.5	-
22:5 n-3	0.3	0.5	0.5	-
22:6 n-3	-	-	16.8	-

Total SFA	40.6	40.7	39.8	9.1
Total MUFA	22.7	18.4	18.0	18.1
Total PUFA	36.8	40.8	42.2	72.8
Total n-6 PUFA	36.0	17.5	20.5	17.7
Total n-3 PUFA	0.7	23.4	21.7	55.1

n-6/n-3 ratio	50.6	0.7	0.9	0.3
PUFA/SFA ratio	0.9	1.0	1.1	8.0

An overlapping of the lines has occurred in the fatty acid profile section of the Table, due to an unfortunate insertion of the 22:2 n-6, a fatty acid that has nothing to do there. This returns any impossible understanding, particularly of the DHA supply and so intake. Table 1 has therefore been replaced here with a version that is both correct and also readable.
